# Role of the CBX Molecular Family in Lung Adenocarcinoma Tumorigenesis and Immune Infiltration

**DOI:** 10.3389/fgene.2021.771062

**Published:** 2021-12-13

**Authors:** Chun Zhang, Lisha Chang, Yizhen Yao, Ce Chao, Zhongchun Ge, Chengfeng Fan, Hualin Yu, Bin Wang, Jingsong Yang

**Affiliations:** ^1^ Department of Oncology, Nanjing First Hospital, Nanjing Medical University, Nanjing, China; ^2^ Department of Oncology, Second Affiliated Hospital, Nanjing Medical University, Nanjing, China; ^3^ Department of Respiratory Medicine, Nanjing Yuhua Hospital, Yuhua Branch of Nanjing First Hospital, Nanjing, China; ^4^ Department of Cardiothoracic Surgery, The Third Affiliated Hospital of Soochow University, Changzhou, China; ^5^ Department of Cardiology, People’s Hospital of Xuyi, Xuyi, China; ^6^ Department of Radiotherapy, Nantong Third People’s Hospital, Nantong University, Nantong, China

**Keywords:** lung adenocarcinoma, CBX molecular family, immune infiltration, tumorigenesis, prognosis

## Abstract

**Background:** The members of the Chromobox (CBX) family are important epigenetic regulatory molecules with critical biological roles in many tumors. However, no study has analyzed or verified their role in lung adenocarcinoma (LUAD).

**Methods:** UALCAN and Oncomine databases were used to analyze CBX expression in LUAD, and the cBioPortal database was used to analyze CBX genetic variations. The Kaplan-Meier plotter and UALCAN databases were used to identify molecules with prognostic value. Gene Ontology pathway, receiver operating characteristic curves, and tumor-infiltrating immune cell analyses were used to clarify the biological function of the CBX hub molecules. Paired tumor samples and lung adenocarcinoma cell lines were collected for molecular functional assays to validate the results of the bioinformatics analysis.

**Results:** CBX3/5 may have a cancer-promoting effect and its expression is associated with a poor patient prognosis, while CBX7 shows an opposite trend. CBX3/5/7 can regulate signaling pathways, regulate tumor immune cell infiltration, and has diagnostic value. Molecular biology experiments show that CBX3/5 is highly expressed in LUAD patients; *in vitro* it promotes the proliferation and migration of the LUAD cell line and can regulate the expression of the corresponding cytokines. CBX7 has opposite effects.

**Conclusion:** Our bioinformatics analysis and subsequent experimental verification confirmed the CBX family members acted as hub signaling molecules in LUAD. The results provide new potential targets for the diagnosis and treatment of this cancer.

## Introduction

Lung adenocarcinoma (LUAD) is one of the deadliest cancers worldwide, and the incidence is increasing annually (Chen et al., 2016). LUAD accounts for more than 40% of all pathological types of lung cancer (Kerdidani et al., 2019). Although there have been many breakthroughs in surgical treatment and immunotherapy approaches, it remains an urgent task to find new targets for LUAD diagnosis and treatment (Hirsch et al., 2017).

CBXs are transcriptional repressors found in normal human cells that regulate cell senescence. Their expression is downregulated in colon cancer, gastric cancer, and hepatocellular carcinoma. As a kind of epigenetic regulatory complex, CBXs repress target gene transcription by modifying chromatin (Ma et al., 2014). The eight known CBX proteins in the human genome are involved in heterochromatin regulation, gene expression, and developmental programming. CBX proteins are divided into two groups according to their molecular structure: The HP 1 group (including CBX1/3/5) and the Pc group (including CBX2/4/6/7/8) (Wotton and Merrill, 2007). Although CBX levels are significantly associated with cancer patient prognosis, there is no consistent pattern among different family members (Liang et al., 2017). CBX1 can serve as a target gene for microRNA-205-5p and thus participates in pituitary tumor progression, while CBX7 regulates stem cell-like properties of gastric cancer cells through the p16 and AKT pathways (Ni et al., 2018; Hu et al., 2019). However, the CBX family has not been studied in depth in LUAD.

Bioinformatics analysis is increasingly performed to stimulate and identify new research directions. This approach was used to elucidate the relationship between LIM domain kinase 1 and immune cell infiltration in LUAD (Lu et al., 2021). Other researchers identified crucial genes and key pathways in breast cancer and Sjögren’s syndrome (Deng et al., 2019; Yao et al., 2019). In this study, we used clinical samples and *in vitro* studies to demonstrate that high expression of CBX3/5 in LUAD tissues promoted tumor cell proliferation and migration of tumor cells and promoted the infiltration of cytotoxic cells, CD8^+^ T cells, and other immune cells. CBX7 has opposite biological functions. Our results provide new potential therapeutic targets for the diagnosis and treatment of LUAD.

## Materials and Methods

### Gene Expression and Survival Analyses

The differential expression of members of the CBX family in LUAD was assessed using the University of Alabama Cancer Database (UALCAN; http://ualcan.path.uab.edu/), Gene Expression Omnibus Database (GEO; https://www.ncbi.nlm.nih.gov/gds/?term=), and the expression of CBX mRNA in multiple types of cancer was analyzed in the Oncomine database (https://www.oncomine.org/resource/login.html) (Chandrashekar et al., 2017; Rhodes et al., 2004), which provides authoritative information on gene expression. Analysis of protein expression levels of members of the CBX family was performed using The Human Protein Atlas Database (http://www.proteinatlas.org/). The association analysis between members of the CBX family and the prognosis of the LUAD patient was explored using the UALCAN and Kaplan-Meier (KM) plotter databases (http://kmplot.com/analysis/) (Menyhart et al., 2018). The two databases were used to applied different survival analysis methods (Kaplan-Meier method and Cox proportional-hazards model).

### Analysis of Genetic Variations

The data from the cBioPortal (http://www.cbioportal.org/) derives from the Cancer Genome Atlas (TCGA) database in the United States, which allows a comprehensive analysis of genomic data from various types of tumors from multiple dimensions (Gao et al., 2013).

### Gene Ontology and Pathway Analyses and Prognostic Signature

We used the org.Hs.eg.db and clusterProfiler R packages for ID conversion and Gene Ontology (GO)+Kyoto Encyclopedia of Genes and Genomes (KEGG) analyses, respectively. Data were visualized using the ggplot2 R package and presented as a bubble graph (Yu et al., 2012; Ito and Murphy, 2013). Log-rank tests were used to compare survival differences obtained from KM analysis between two or more groups, and time-dependent receiver operating characteristic (ROC) curve analyses were performed to compare gene signature prediction accuracy and risk score. The area under the curve (AUC) was defined as the area under the ROC curve. AUC values were used as evaluation criteria for diagnostic models. The least absolute shrinkage and selection operator (LASSO) regression algorithm was used for feature selection with a 10-fold cross-validation using the glmnet R package.

### Co-Expressed Gene Analyses and Tumor-Infiltrating Immune Cells

The STAT R package was used to analyze genes co-expressed with CBX3/5/7. The screening threshold was a Pearson’s correlation coefficient >0.5. These data were visualized using the ggplot2 R package and are presented as a heatmap.

Patients were divided into two groups according to the median expression values of CBX3/5/7 and then the GSVA R package and a single-sample gene set enrichment analysis (ssGSEA) immune infiltration algorithm was used to compare immune cell infiltration between two groups. The data are presented as box plots. The *p*-values were obtained by the Wilcoxon rank sum test.

### Cell Lines and Clinical Samples

Human LUAD A549, SPCA1, H1299, and H1975 cell lines were cultured in Dulbecco’s minimum essential medium supplemented with 10% fetal bovine serum (FBS; GIBCO, Grand Island, NY, United States) and 1% penicillin/streptomycin solution at 37°C in a cell incubator.

Thirty-six LUAD and paired normal tissue samples were obtained from patients who underwent surgery at the Third Affiliated Hospital of Soochow University. None of the included patients had undergone radiation, chemotherapy, or other preoperative treatment. This study complied with the Ethics Code of the World Medical Association set out in the Declaration of Helsinki. Studies involving human participants were reviewed and approved by the Ethics Committee of the Third Affiliated Hospital of Soochow University (IRB number: (2021) 005). All patients provided their written informed consent to participate in this study.

### Cell Transfection

Small interfering RNA (siRNA) targeting CBX3, CBX5, and Negative control (NC) were purchased from GenePharma (Shanghai, China), and were transfected into LUAD cell lines using Lipofectamine 3000 (Invitrogen, Carlsbad, CA, United States) according to the manufacturer’s instructions. CBX7 cDNA was cloned into the pcDNA3.1+ vector purchased from Genebay Biotech (Nanjing, China) and then transfected into LUAD cell lines using Lipofectamine 3000.

### RNA Extraction, Quantitative Polymerase Chain Reaction and ELISA

Tissue (100 mg) or cells were ground to powder in liquid nitrogen, 1 ml of TRIzol was added to fully lyse the cells, and RNA was extracted. The PrimerScript Reverse Transcription Kit (RR047A, Takara, Shiga, Japan) was used for cDNA preparation and the TB Green™ Premix Ex Taq (RR420A, Takara) was used for qPCR. Relative gene expression levels were analyzed using the 2^−ΔΔCt^ method. interleukin (IL)-6, or interferon (IFN)-γ in culture supernatants of LUAD cell line were quantified using IL-6/IFN-γ Quantikine ELISA kit (R&D Systems, CA, United States) according to the manufacturer’s instructions.

### Cell Proliferation Assay

The proliferative ability of the cell lines was examined using the Cell Counting Kit-9 (CCK8) assays. Cells were seeded in a 96-well plate (2.0 × 10^3^ cells/well) and 10 μl of CCK8 reagent (Dojindo, Kumamoto, Japan) was added to each well at five time points (0, 24, 48, 72, and 96 h). After 1 h of incubation in a cell incubator at 37°C, the optical density of the sample (OD) at 450 nm was detected using a microplate reader.

### Transwell Migration Assay

Transwell inserts were inserted into a 24-well plate and 200 μl of the cell suspension (4.0 × 10^4^ cells/well, resuspended in serum-free culture medium) was added to the upper chamber and 800 μl of medium containing 20% FBS was added to the lower chamber. After incubation for 24 h, cells that had not migrated in the upper chamber were removed. After fixation and crystal violet staining, photographs were taken under a microscope.

### Statistical Analysis

GraphPad Prism 8 software (GraphPad Inc., San Diego, CA, United States) was used to perform a one-way analysis of variance analysis and paired Student’s t-tests to analyze differences between multiple groups or two groups, respectively.

## Results

### Dysregulated Expression of CBXs in LUAD

**FIGURE 1:**
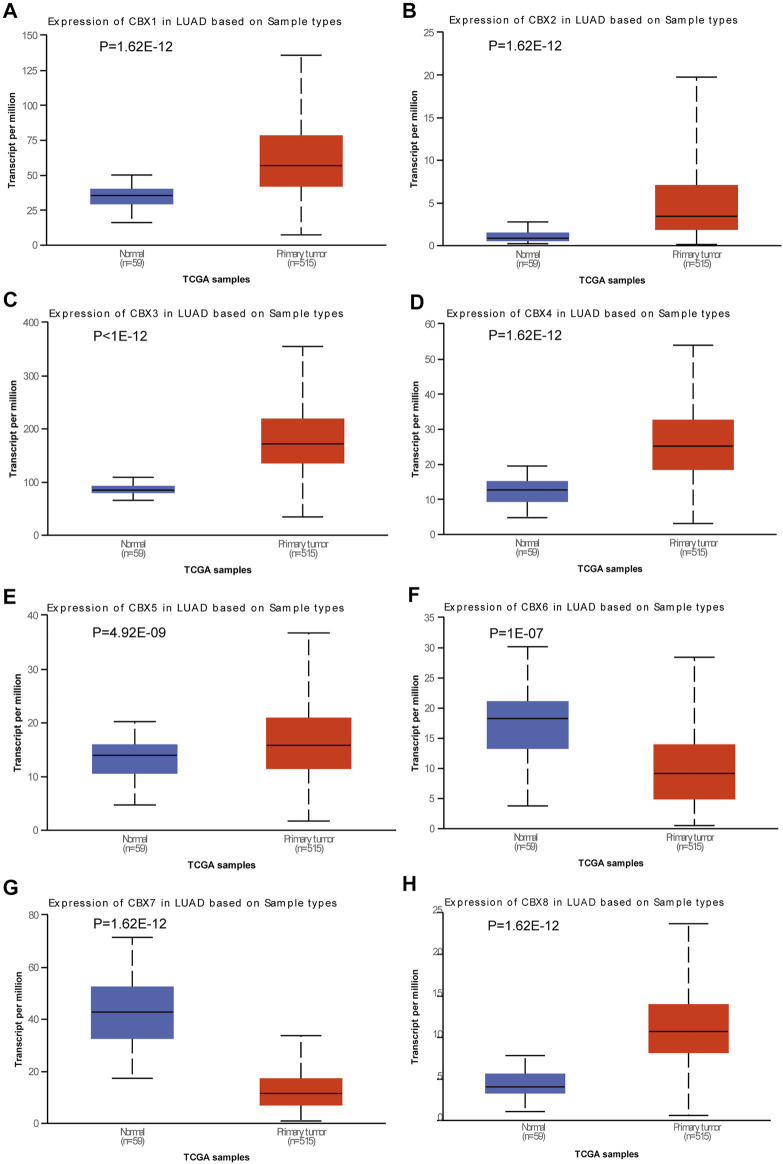
Expression of CBX family members in lung adenocarcinoma (LUAD). Box plots showing the expression of CBX family members in LUAD compared to normal tissues. **(A)** CBX1, **(B)** CBX2, **(C)** CBX3, **(D)** CBX4, **(E)** CBX5, **(F)** CBX6, **(G)** CBX7, and **(H)** CBX8. Differences were considered significant at *p* < 0.05.

**FIGURE 2:**
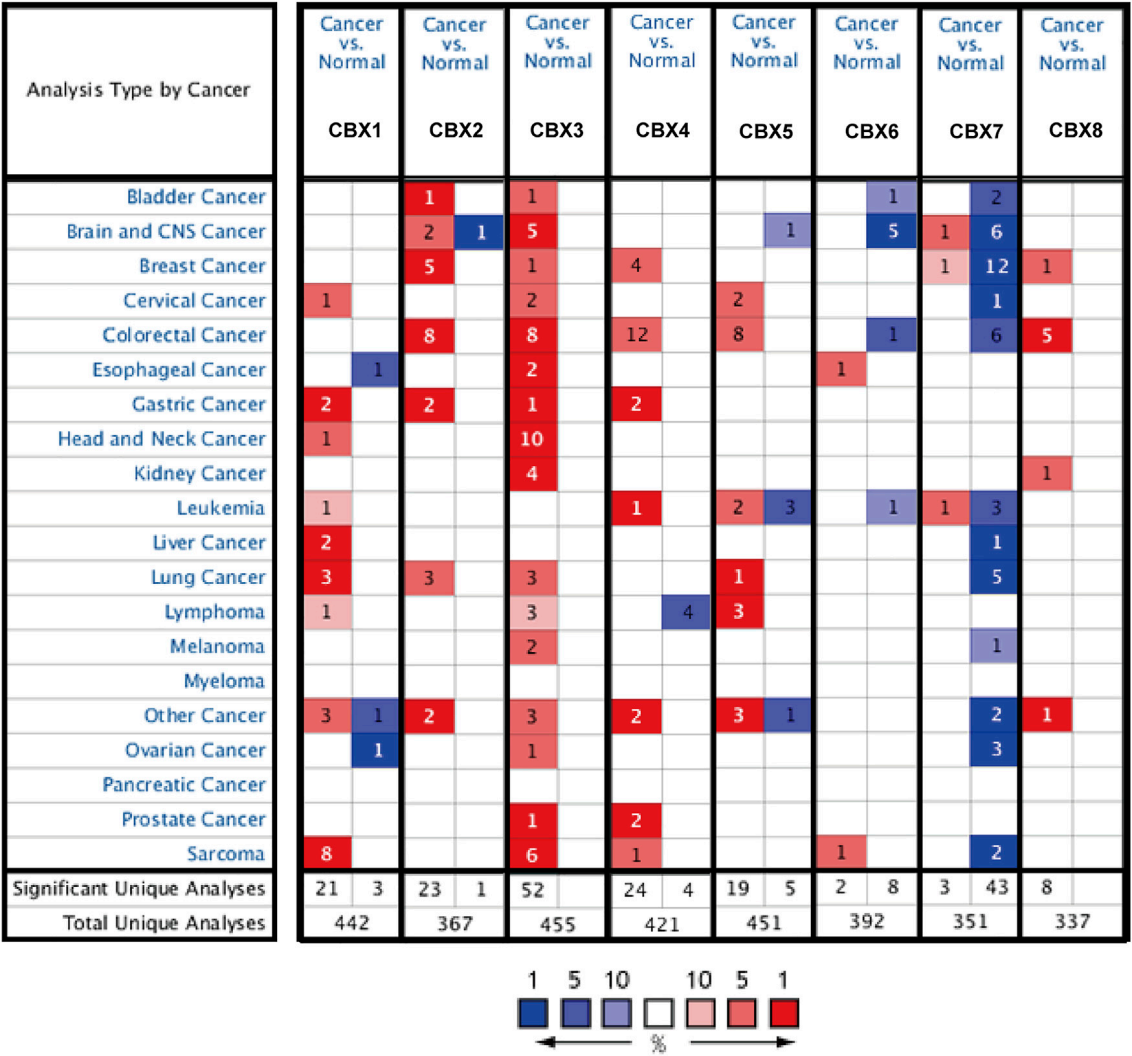
CBX mRNA expression levels in pan-cancer. The number indicates the number of data sets validated in the Oncomine database for the differential expression of each gene. Red and blue squares indicate that gene expression is up- or downregulated, respectively.

We used the UALCAN database to explore the expression of members of the CBX family in LUAD and found significant differences between them (Figures 1A–H). To ensure the reliability of the results, we also used the lung adenocarcinoma data set (GSE10072) in the GEO database for further validation. Besides CBX2, the expression trend of other molecules of the CBX family was consistent with the conclusion obtained by using UCLCAN database (Supplementary Figure S1). Molecules with widely dysregulated expression in various tumors are likely to play an important role in tumorigenesis, so we also queried the Oncomine database to explore the expression of CBXs in various tumor types. We found that CBXs showed a consistent trend of differential gene expression in various cancers including lung cancer, colorectal cancer, and breast cancer (Figure 2). In lung cancer, the expression of CBX1, CBX2, CBX3, and CBX5 was up-regulated, while CBX7 was down-regulated (Figure 2). The importance of the CBX family of molecules was further underlined by this pan-cancerous property.

### Analysis of CBX Gene Mutations in LUAD

**FIGURE 3:**
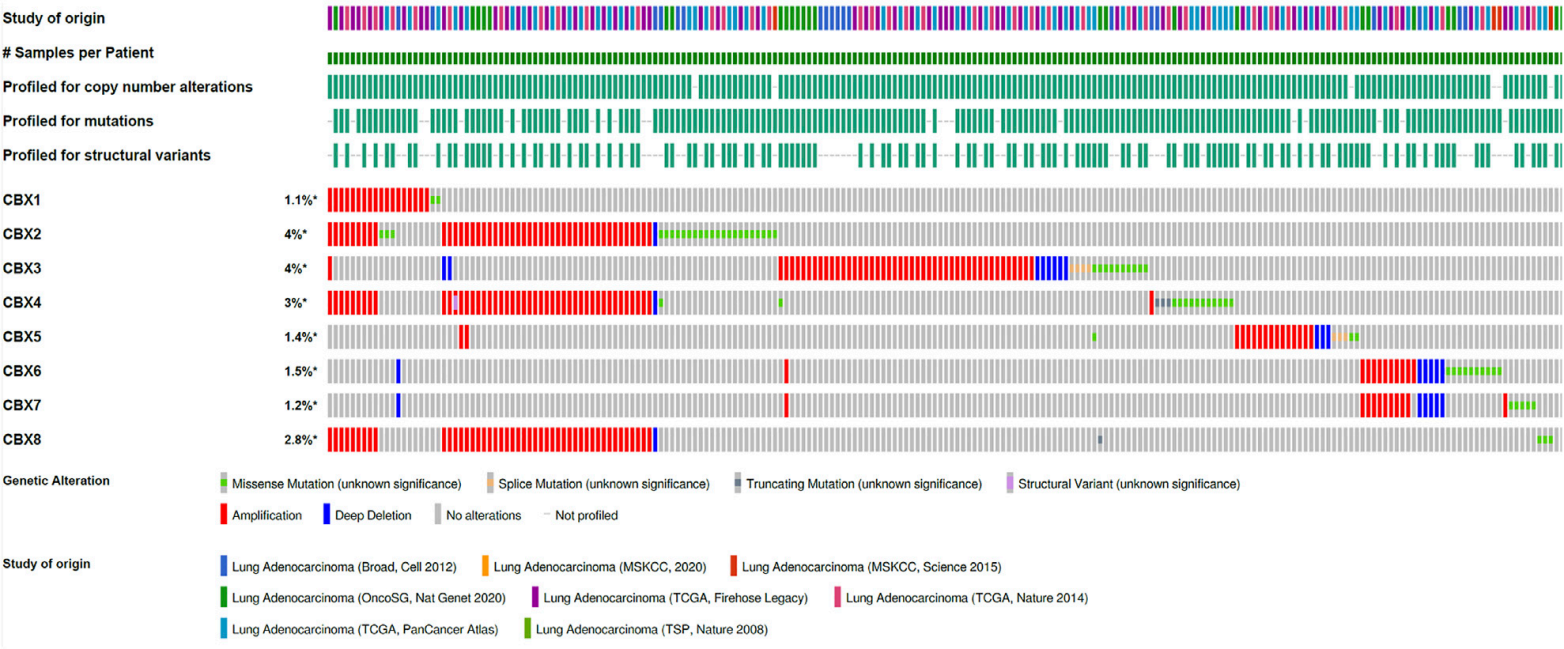
Amplification and mutation of CBX family members in LUAD. Genomic variations of CBX family members in 3098 cases were analyzed using the cBioPortal database.

Genetic variants are also important for tumorigenesis, so CBX gene variants were analyzed using the cBioPortal database (Figure 3). We found varying degrees of genetic variations in the eight known molecules. These common genomic changes included gene amplification and gene deletion, which are considered important tumorigenesis factors. Most CBXs were amplified, deleted, and mutated in LUAD, with the highest incidence rates involving CBX3 and CBX4.

### Analysis of Prognosis and Clinical Correlation of CBX Expression in Patients with LUAD

**FIGURE 4:**
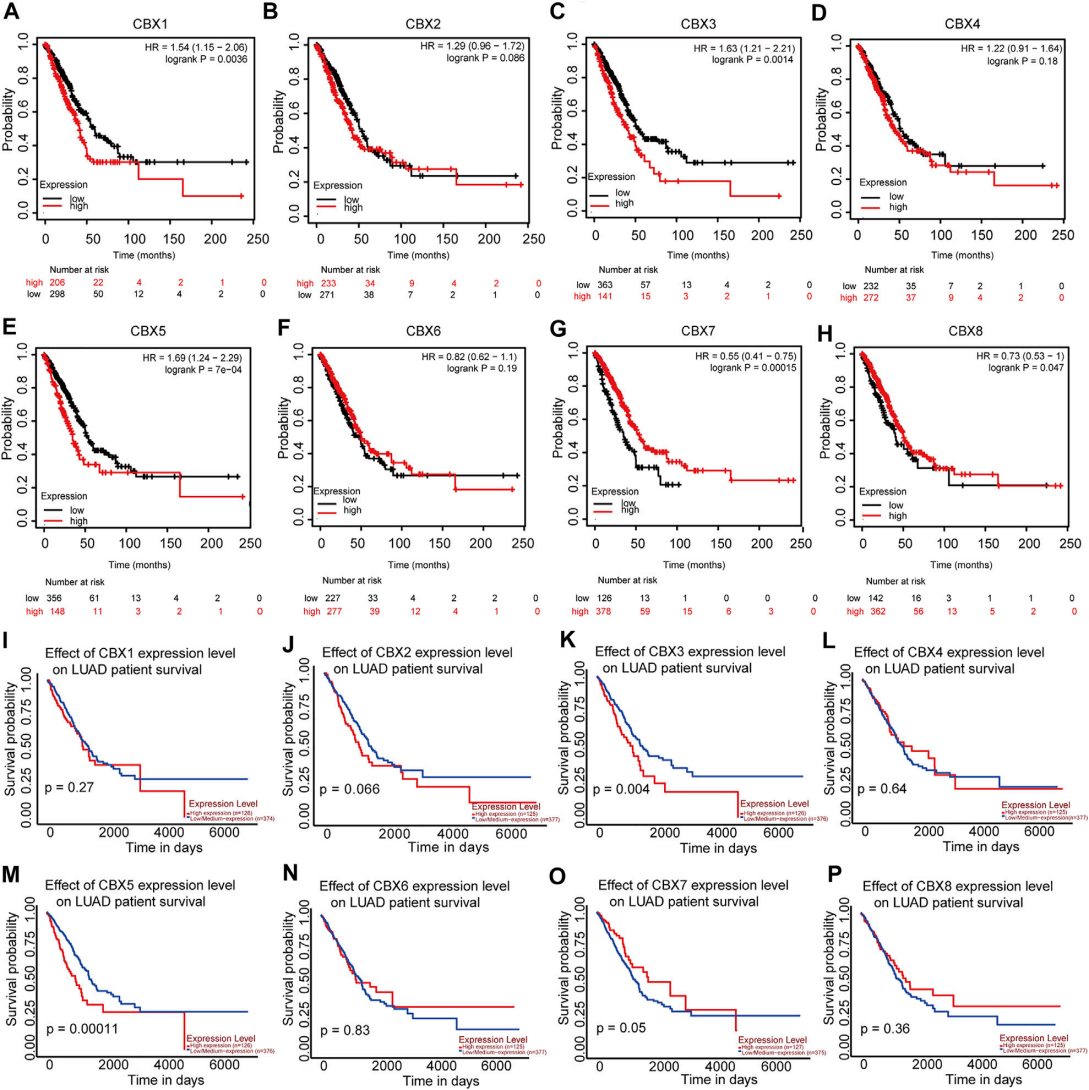
CBX1/3/5/7/8 correlations with LUAD prognosis. **(A–H)** Correlations between CBX1/3/5/7/8 and overall survival status in patients with LUAD (KM) and **(I–P)** in patients with LUAD (UALCAN).

Considering the importance of molecular prognostic markers, we used the KM plotter database to explore the predictive utility of CBXs in LUAD. CBX1, CBX3, and CBX5 were associated with poor prognosis (Figures 4A,C,Efig4), while CBX7 and CBX8 showed the opposite trend (Figures 4G,H). Similarly, we also evaluated the prognostic value of these molecules in the UALCAN database, which showed that CBX3 and CBX5 were associated with a poor prognosis, while CBX7 was associated with a good prognosis in patients with LUAD (Figures 4K,M,O). The method of survival analysis used in these two databases differed, thus taking the intersection of the results from the two databases was used to eliminate statistical bias. Overall, the differences in expression and prognostic values of CBX3, CBX5, and CBX7 were consistent among different databases. To further clarify the roles of CBX3, CBX5, and CBX7 in disease progression, we use the UALCAN database to analyze CBX expression in different patients with different disease grades and lymph node metastasis status. The results showed there was a trend for higher expression of CBX3 and CBX5 with the progression of LUAD, in terms of worse tumor grade, while the expression of CBX7 showed a decreasing trend (Supplementary Figures S2A–C). The same pattern was observed for patients with more severe lymph node metastasis (Supplementary Figures S2D–F). We then downloaded relevant LUAD data from TCGA database and performed an association analysis in terms of the expression of these three molecules. The results showed that CBX3 was positively correlated with T stage and N stage (Table 1); CBX5 was positively correlated with M stage and pathology stage (Table 2); and CBX7 was negatively correlated with the T stage, N stage and pathology stage (Table 3). Collectively, these results suggested that among members of the CBX family, CBX3/5/7 may have the strongest impact on LUAD.

**TABLE 1:** Correlation between CBX3 expression and clinicopathologic characteristics of LUAD patients (n = 535).

Characteristic	Low expression of CBX3	High expression of CBX3	*p*
n	267	268	
T stage, n (%)			0.032
T1	100 (18.8%)	75 (14.1%)	
T2	132 (24.8%)	157 (29.5%)	
T3	27 (5.1%)	22 (4.1%)	
T4	6 (1.1%)	13 (2.4%)	
N stage, n (%)			0.013
N0	187 (36%)	161 (31%)	
N1	43 (8.3%)	52 (10%)	
N2	27 (5.2%)	47 (9.1%)	
N3	0 (0%)	2 (0.4%)	
M stage, n (%)			0.343
M0	173 (44.8%)	188 (48.7%)	
M1	9 (2.3%)	16 (4.1%)	
Pathologic stage, n (%)			0.205
Stage I	154 (29.2%)	140 (26.6%)	
Stage II	64 (12.1%)	59 (11.2%)	
Stage III	35 (6.6%)	49 (9.3%)	
Stage IV	10 (1.9%)	16 (3%)	
Gender, n (%)			0.631
Female	146 (27.3%)	140 (26.2%)	
Male	121 (22.6%)	128 (23.9%)	
Smoker, n (%)			0.422
No	41 (7.9%)	34 (6.5%)	
Yes	218 (41.8%)	228 (43.8%)	
Age, n (%)			0.034
≤65	114 (22.1%)	141 (27.3%)	
>65	142 (27.5%)	119 (23.1%)	
Age, meidan (IQR)	67 (59, 74)	65 (59, 71.25)	0.011

**TABLE 2:** Correlation between CBX5 expression and clinicopathologic characteristics of LUAD patients (n = 535).

Characteristic	Low expression of CBX5	High expression of CBX5	*p*
n	267	268	
T stage, n (%)			0.066
T1	86 (16.2%)	89 (16.7%)	
T2	151 (28.4%)	138 (25.9%)	
T3	26 (4.9%)	23 (4.3%)	
T4	4 (0.8%)	15 (2.8%)	
N stage, n (%)			0.977
N0	174 (33.5%)	174 (33.5%)	
N1	49 (9.4%)	46 (8.9%)	
N2	38 (7.3%)	36 (6.9%)	
N3	1 (0.2%)	1 (0.2%)	
M stage, n (%)			0.010
M0	190 (49.2%)	171 (44.3%)	
M1	6 (1.6%)	19 (4.9%)	
Pathologic stage, n (%)			0.044
Stage I	151 (28.7%)	143 (27.1%)	
Stage II	64 (12.1%)	59 (11.2%)	
Stage III	40 (7.6%)	44 (8.3%)	
Stage IV	6 (1.1%)	20 (3.8%)	
Gender, n (%)			0.129
Female	152 (28.4%)	134 (25%)	
Male	115 (21.5%)	134 (25%)	
Age, n (%)			0.860
≤65	126 (24.4%)	129 (25%)	
>65	132 (25.6%)	129 (25%)	
Smoker, n (%)			0.957
No	38 (7.3%)	37 (7.1%)	
Yes	221 (42.4%)	225 (43.2%)	
Age, meidan (IQR)	66 (59, 72)	65.5 (59, 72)	0.847

**TABLE 3:** Correlation between CBX7 expression and clinicopathologic characteristics of LUAD patients (n = 535).

Characteristic	Low expression of CBX7	High expression of CBX7	*p*
N	267	268	
T stage, n (%)			<0.001
T1	63 (11.8%)	112 (21.1%)	
T2	166 (31.2%)	123 (23.1%)	
T3	24 (4.5%)	25 (4.7%)	
T4	13 (2.4%)	6 (1.1%)	
N stage, n (%)			0.001
N0	162 (31.2%)	186 (35.8%)	
N1	47 (9.1%)	48 (9.2%)	
N2	52 (10%)	22 (4.2%)	
N3	1 (0.2%)	1 (0.2%)	
M stage, n (%)			0.991
M0	194 (50.3%)	167 (43.3%)	
M1	14 (3.6%)	11 (2.8%)	
Pathologic stage, n (%)			0.004
Stage I	134 (25.4%)	160 (30.4%)	
Stage II	61 (11.6%)	62 (11.8%)	
Stage III	57 (10.8%)	27 (5.1%)	
Stage IV	14 (2.7%)	12 (2.3%)	
Gender, n (%)			0.052
Female	131 (24.5%)	155 (29%)	
Male	136 (25.4%)	113 (21.1%)	
Age, n (%)			0.005
≤65	145 (28.1%)	110 (21.3%)	
>65	115 (22.3%)	146 (28.3%)	
Smoker, n (%)			0.006
No	26 (5%)	49 (9.4%)	
Yes	234 (44.9%)	212 (40.7%)	
Age, meidan (IQR)	63 (57.75, 71)	67 (60, 74)	<0.001

### GO and Pathway Analyses of Co-Expressed Genes of CBX3/5/7

**FIGURE 5:**
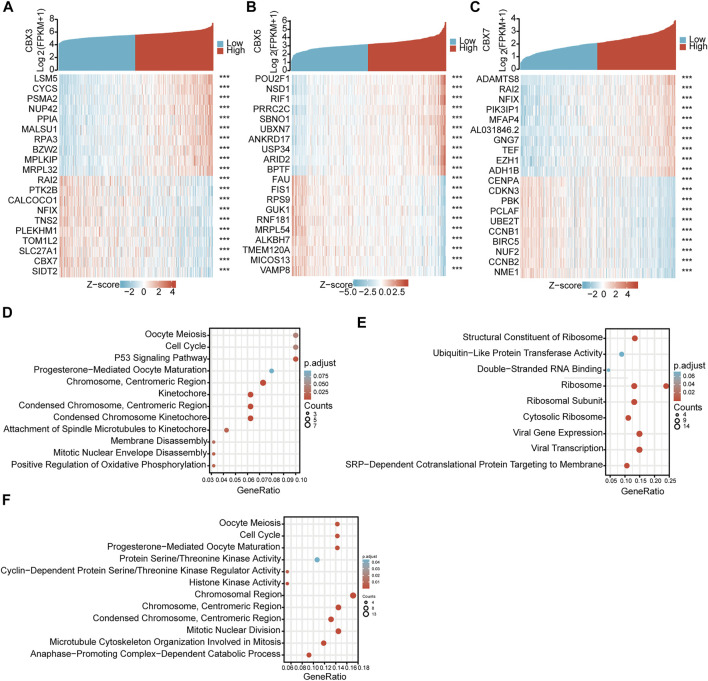
GO and Pathway enrichments of CBX3/5/7 co-expressed genes. **(A)** Co-expressed genes of CBX3, **(B)** CBX5, and **(C)** CBX7. **(D–F)** Bioinformatics analysis of CBX3, CBX5, and CBX7 co-expressed genes in LUAD. ***p < 0.001.

To clarify the mutual regulatory relationships, we analyzed genes co-expressed with these three CBX molecules. By mining and analyzing the transcriptome data of TCGA-LUAD, we identified 437 positively and 72 negatively correlated genes for CBX3 (Figure 5A), 65 positively and 379 negatively correlated genes for CBX5 (Figure 5B), and 365 positively and 28 negatively correlated genes for CBX7 (Figure 5C). These co-expressed genes potentially reflect the biological functions of CBX3, CBX5, and CBX7. Thus, we performed GO and KEGG pathway analyses, which revealed that CBX3 was related to cell cycle regulation and P53 signaling pathways (Figure 5D), CBX5 was related to ubiquitin-like protein transferase activity and ribosome pathways (Figure 5E), while CBX7 was related to the cell cycle and cyclin-dependent protein serine/threonine kinase regulator activity pathways (Figure 5F).

### The Prognostic Value of CBX3/5/7

**FIGURE 6:**
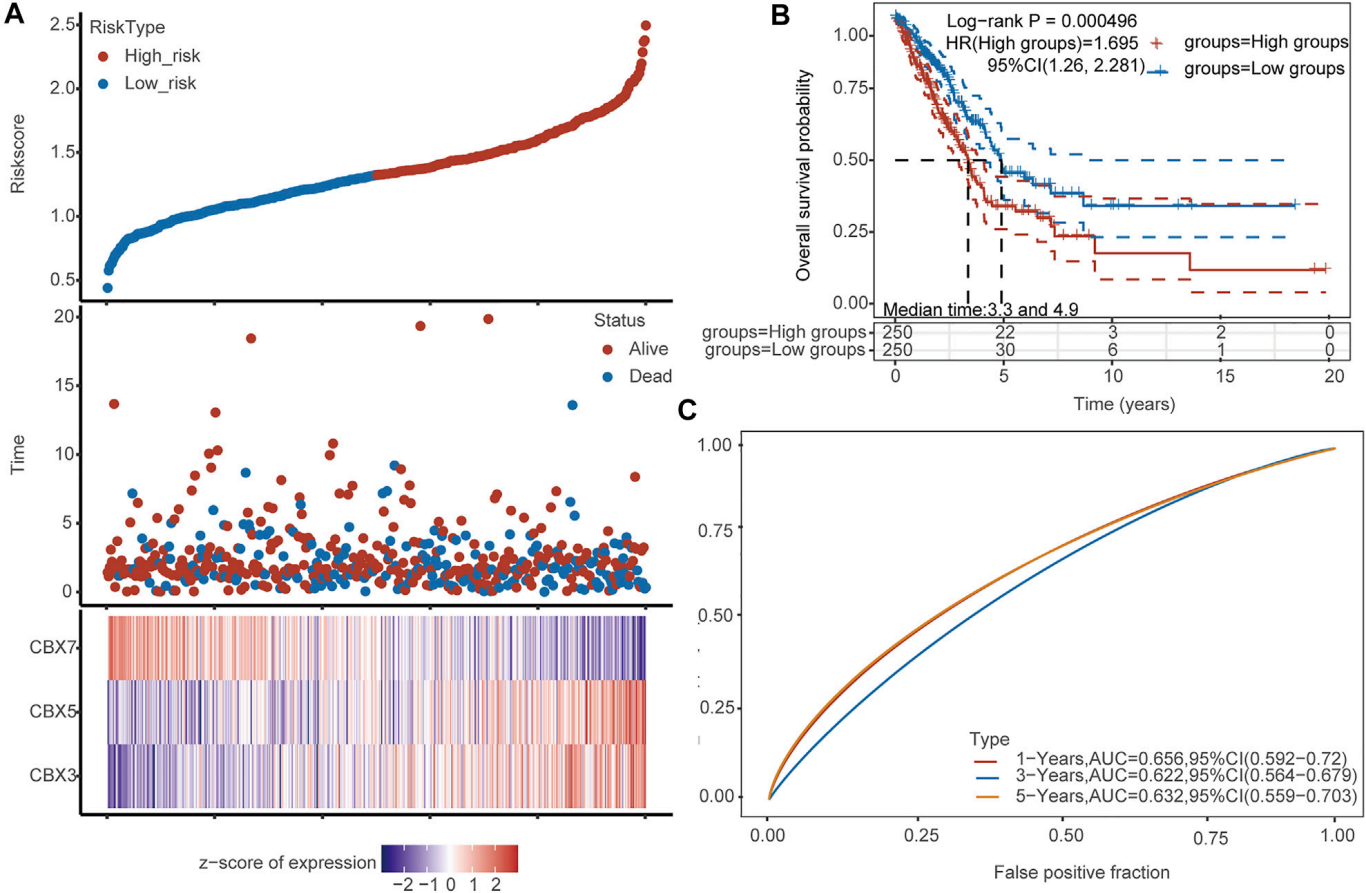
Analysis of the diagnostic value of CBX3/5/7. **(A)** Patients were divided into low- and high-risk groups according to the median risk score. Heatmap of the expression profiles of the prognostic genes in the low- and high-risk groups. **(B)** Both groups of LUAD patients have significant prognostic differences. **(C)** Time-dependent ROC analysis based on the CBX signature.

To verify the predictive prognostic efficacy of CBX3, CBX5, and CBX7, we used TCGA datasets and performed LASSO regression to reduce dimensionality and build a prognostic model based on CBX3, CBX5, and CBX7 expression (lambda.min = 0.0023; Risk score = (0.1442) × CBX3 + (0.2588) × CBX5 + (−0.2601) × CBX7). Patients were divided into low- and high-risk groups according to the median risk score. The heatmap of the expression profiles of prognostic genes in low- and high-risk groups is shown in Figure 6A. LUAD patients were also divided into two groups using our molecular signature, and the results showed significant prognostic differences (Figure 6B), confirming the effectiveness of the model. Time-dependent ROC analysis (5-years, AUC = 0.632, 95%CI: 0.559–0.703) based on the three-gene signature confirmed that our model had little clinical value in the diagnosis of LUAD (Figure 6C).

### Correlation Analysis of CBX3/5/7 Expression and Infiltrating Immune Cells in LUAD

**FIGURE 7:**
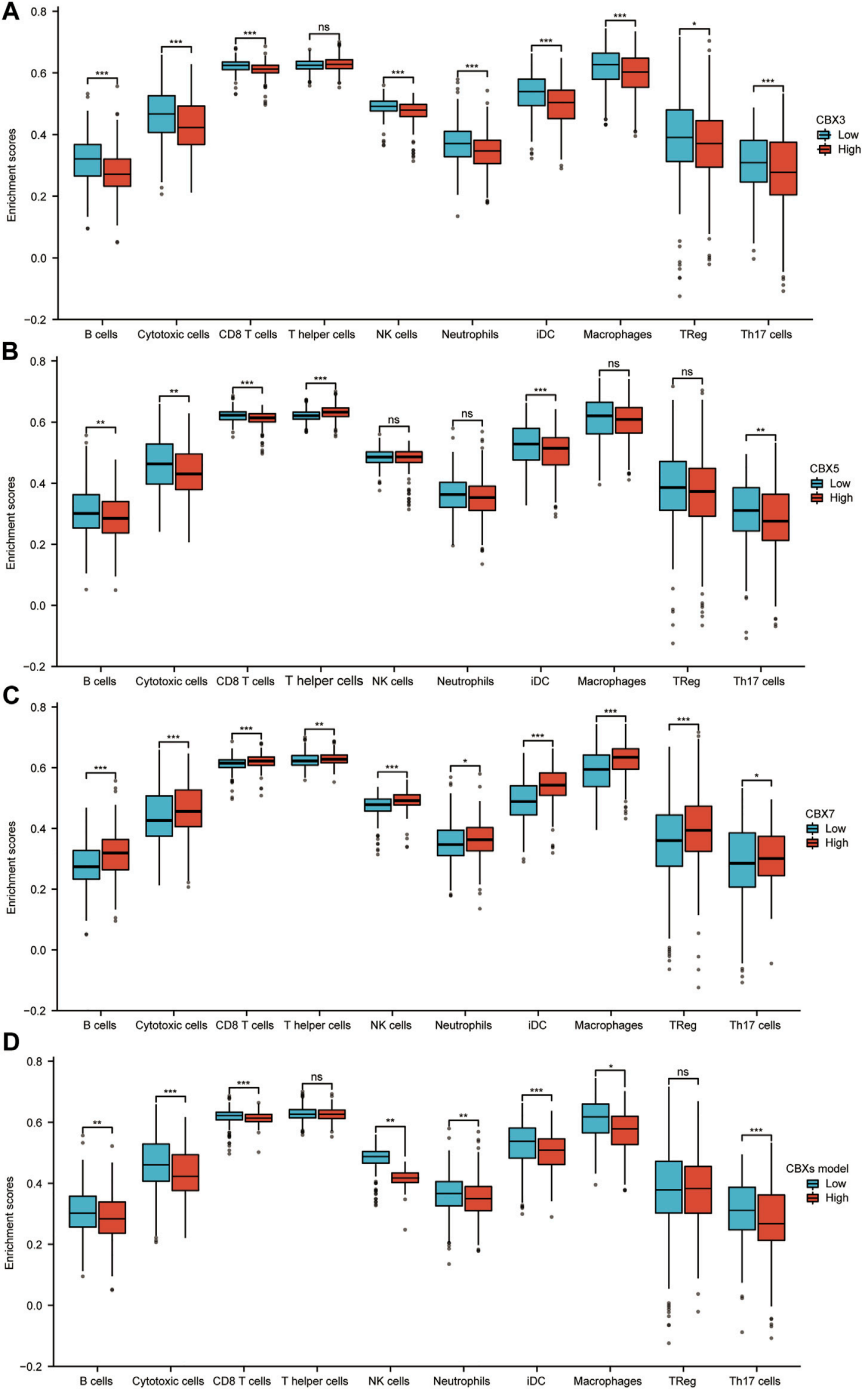
Correlation analysis of CBX3/5/7 expression and infiltration levels of immune cells in LUAD tissues. **(A–D)** Box plots of the correlations between CBX3/5/7 or molecular model expression and infiltration levels of immune cells. *p < 0.05, **p < 0.01, ***p < 0.001.

The immune microenvironment is currently a research hotspot. Therefore, we analyzed the relationship between CBX3/5/7 expression levels and infiltrating immune cells in LUAD. Patients in the CBX3 high-expression group (n = 268, data from TCGA database) showed a reduction in the numbers of infiltrating B cells, cytotoxic cells, CD8^+^ T cells, natural killer (NK) cells, neutrophils, and macrophages (Figure 7A). Patients in the high expression CBX5 group also showed a decrease in immune cell infiltration, including B cells, cytotoxic cells, CD8^+^ T cells, and Th17 cells (Figure 7B). The group with higher expression of CBX7, associated with an inhibition of tumor progression, presented larger numbers of infiltrating immune cells (Figure 7C). In tumor immune microenvironments with high expression of CBX7, the infiltration of immune cells able to kill tumor cells increased, including B cells, cytotoxic cells, CD8^+^ T cells, NK cells, and macrophages (Figure 7C). According to the results of our molecular prediction model, we also explored the infiltration of immune cells in the high-expression group and the low-expression group of the CBX3/5/7 molecular model. The results suggested that our combined model may be better suited to assess the outcome of tumor immune cell infiltration than either molecule alone. In view of the role of CBX3/5/7 in tumor growth-related signaling pathways, the tumor immune microenvironment, and patient prognosis, we believe that CBX3/5/7 may be hub molecules of the CBX molecular family.

### CBX3/5 Promoted and CBX7 Inhibited the Proliferation and Migration of LUAD Cell Lines

**FIGURE 8:**
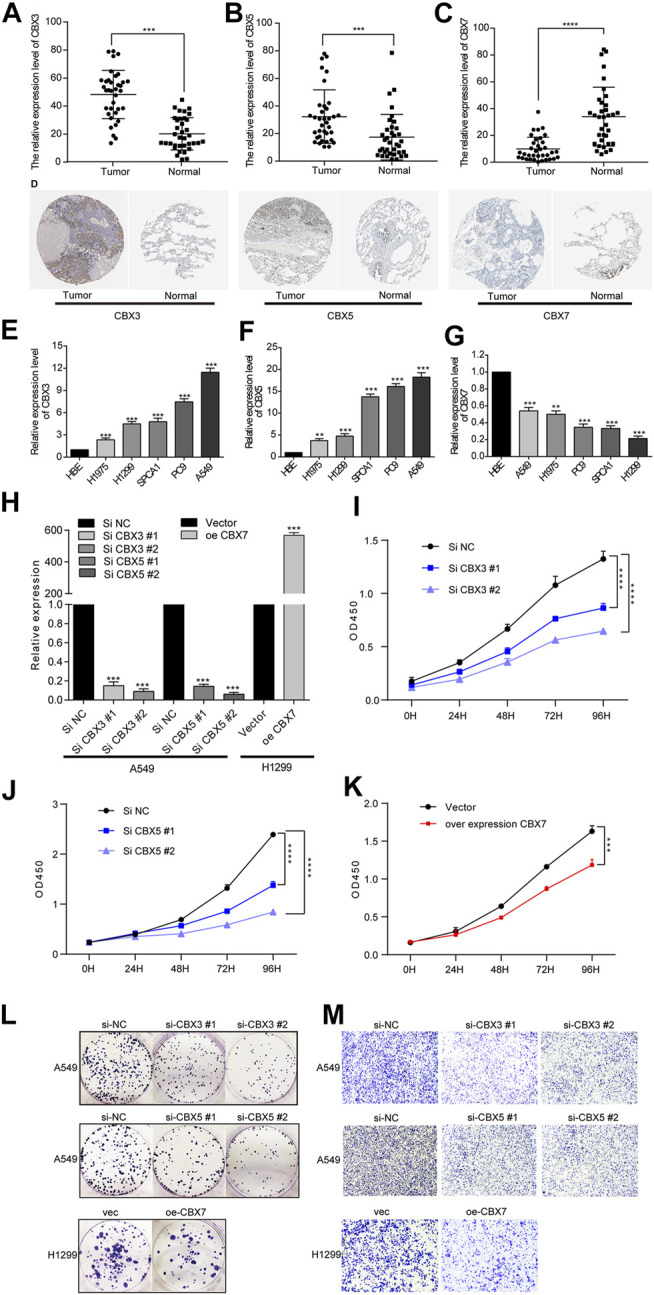
Opposing expression trends and biological effects of CBX3/5 and CBX7 in LUAD tissues or cultured cells. **(A–C)** Opposing expression trends of CBX3/5 and CBX7 in LUAD tissues compared with normal tissues using qPCR analysis. **(D)** Representative picture of CBX3/5/7 protein expression trend **(E–G)** CBX3/5/7 expression in LUAD cell lines. **(H)** Verification of CBX3/5/7 overexpression and knockdown efficiency in LUAD cell lines. **(I–K)** CCK-8 cell proliferation assay results from LUAD cells. **(L and M)** Clone formation and Transwell assay results from LUAD cells. NC: negative control. *p < 0.05, **p < 0.01, and ***p < 0.001.

To verify the above results, we collected 36 matched normal and LUAD tissues to measure CBX3/5/7 expression. qPCR detection confirmed that CBX3/5 and CBX7 were up- and down-regulated in LUAD, respectively (Figures 8A–C). Then we used the Human Protein Atlas Database to obtain the protein expression of CBX3/5/7 in matched normal and LUAD tissues, and the results were consistent with the RNA expression. Figure 8D is a typical representative image. Next, we elucidated the biological effects of CBX3/5/7 through *in vitro* studies. We initially selected five lung adenocarcinoma cell lines to detect the expression of CBX3/5/7 by qPCR. Considering the transfection efficiency and gene expression profiles, we used the A549 and H1299 cell lines for the subsequent experiments (Figures 8E–G). First, we performed qPCR to verify the knockdown and overexpression efficiencies of CBX3/5 and CBX7, respectively (Figure 8H). The cell proliferation ability was then measured using clone formation and CCK-8 assays. Our experiments confirmed that CBX3/5 promoted the proliferation and migration of the LUAD cell lines, effects which were inhibited by CBX7 (Figures 8I–L and Figure 8M).

### CBX3/5 May Regulate the Immune Microenvironment and Cell Cycle Transition in Lung Adenocarcinoma

**FIGURE 9:**
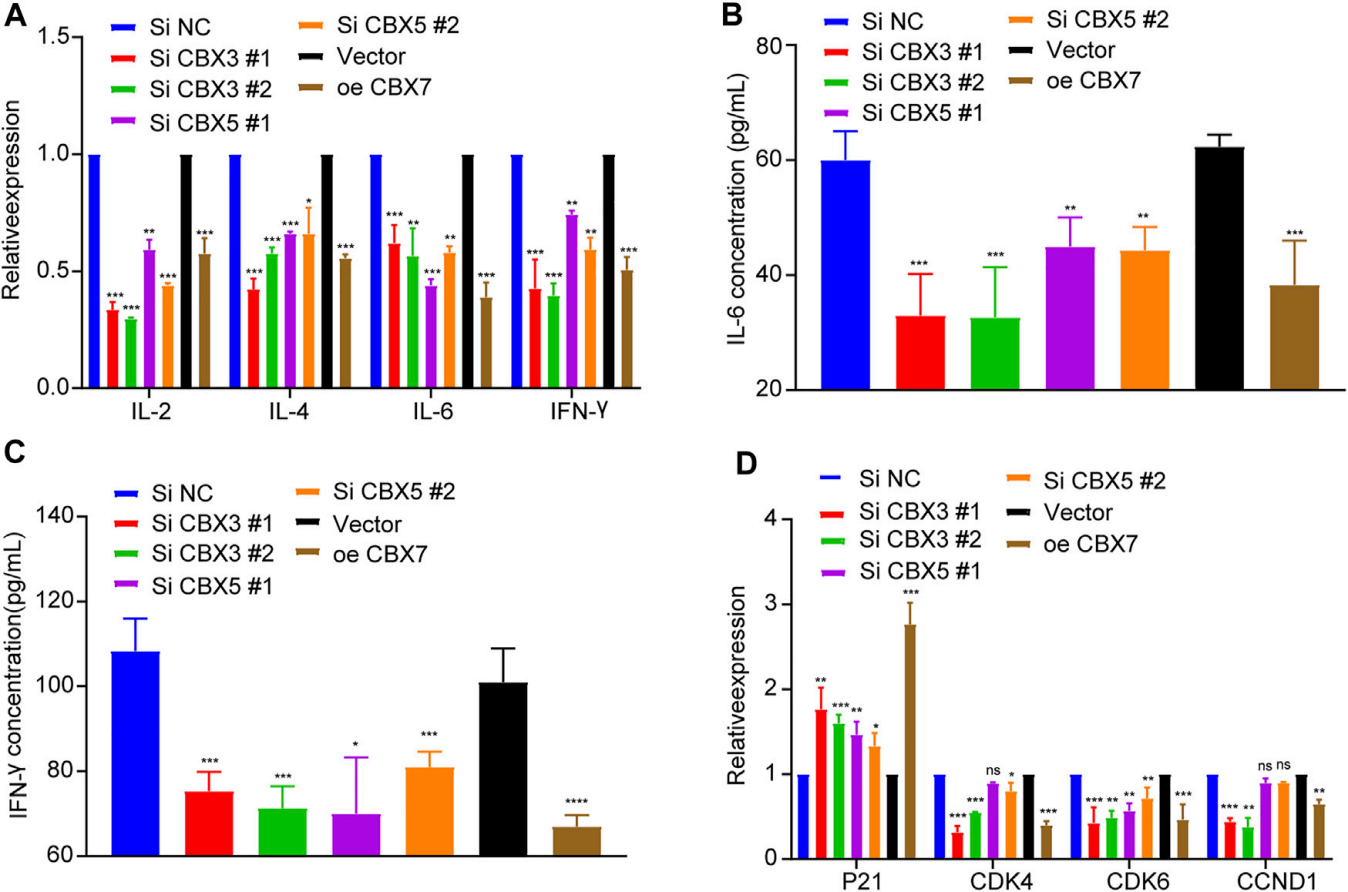
CBX3/5/7 regulates the immune microenvironment and cell cycle transition in LUAD. **(A)** Cytokine expression and **(B and C)** cytokine secretion levels in LUAD cells. **(D)** Cell cycle transition related molecules expression levels in LUAD cells. NC: negative control. NS = Not Statistically Significant. *p < 0.05, **p < 0.01, and ***p < 0.001.

To further explore the effects of CBX3/5/7 on the tumor immune microenvironment, we used qPCR to detect cytokine expression after molecular manipulation. CBX3/5 knockdown inhibited the expression of IL-2, IL-4, IL-6, and IFN-γ, and CBX7 overexpression yielded similar results (Figure 9A). This indicated there CBX3/5/7 exerted potential effects on the tumor microenvironment and further illustrates its important position within the CBX molecular family. Cytokines can be secreted by tumor cells and may regulate the tumor immune microenvironment. The ELISA assay findings demonstrated that CBX3/5 promoted and CBX7 inhibited the secretion of IL-6 and IFN-γ in lung adenocarcinoma cell lines (Figures 9B,C). The cell cycle transition is an important step in the division and proliferation of tumor cells. According to the results of gene enrichment, CBX3 and CBX7 can regulate cell cycle transition. We also confirmed that CBX3/7 could regulate four cell cycle transition-related molecules (P21, CDK4, CDK6, CCND1) by q-PCR. Of note, CBX5 was also able to regulate P21 and CDK6, two of four molecules related to the cell cycle (Figure 9D).

## Discussion

Lung cancer is the most common cancer in China and one of the leading causes of cancer-related deaths. At present, imaging and tumor marker detection are the main non-invasive screening methods of lung cancer used to evaluate progression and monitor therapeutic effects. However, conventional computed tomography has low sensitivity for early lung cancer and recurrent metastases. The serum tumor markers carcinoembryonic antigen and neuron-specific enolase have a certain role in the diagnosis of lung cancer and can also be used as indicators of tumor recurrence after surgical intervention and chemotherapy; however, they have high false positive rates due to benign tumors, pregnancy, chronic inflammatory diseases, or other factors, reducing their reliability (Jiang et al., 2018). LUAD accounts for more than 40% of all types of lung cancer (Kerdidani et al., 2019). Therefore, exploring molecules that promote or inhibit the occurrence of LUAD and identifying more appropriate and noninvasive biomarkers to predict the occurrence or prognosis is important for guiding effective clinical treatment.

Several studies have reported that CBX plays a key role in cancer occurrence and development (Li et al., 2014; Liang et al., 2017; Zhong et al., 2019; Jiang et al., 2020), but various members of the CBX family may play different roles in lung cancer pathogenesis. Nonetheless, conflicting data complicate the significance of CBX as a lung cancer biomarker. Therefore, we used the large lung cancer datasets from CBX and TCGA for our bioinformatics analysis bioinformatics. LUAD tissues also collected for further verification, and finally functional cell experiments were carried out to comprehensively reveal the hub position of CBX3/5/7 in the CBX molecular family, as well as to clarify its biological role and prognostic value in LUAD.

Our study is the first to analyze the differential expression of eight molecules of members of the CBX family through the UALCAN database. We found that the CBX molecule family is widely dysregulated in LUAD, which was expected as the data of UALCAN database derives from TCGA database. The content of a single database is not sufficient to reliably sustain the results. It is more convincing to derive the same results querying different datasets. Thus, we extracted data from a lung adenocarcinoma microarray dataset (GSE10072) from the GEO database. The results showed that the expression trend of other molecules was consistent with those from the TCGA except for CBX2. If a molecule has a consistent expression trend in various types of tumors, it indicates that it has pan-cancer characteristics and may play a strong role in the regulation of tumorigenesis. Therefore, we also used the Oncomine database to analyze the expression of members of the CBX family in different types of cancer. The results showed that CBX1/2/3/5/7 are dysregulated in lung cancer and show a unified trend in cervical cancer and colorectal cancer. Unfortunately, the Oncomine database does not separate lung cancer according to pathological type, i.e., lung adenocarcinoma and lung squamous cell carcinoma. This also led us to collect clinical samples from our own patients for verification. In general, CBX1/2/3/5/7 have the greatest potential and merit study among the members of the CBX family. We also investigated modifications in the CBX family members at the genomic level. Interestingly, CBX7 was not associated with any deletions and mutations, despite its mRNA level being lower in LUAD tumor tissues. A possible reason may be that of histone modification, which is responsible for regulating the degree of chromatin compaction, which thereby interferes with the binding of transcription factors (Qian and Zhou, 2006). There may be some negatively regulated histone modifications that inhibit the transcription of CBX7. Transcription factors can be used as an important element to regulate gene expression, the expression level of transcription factors regulating the expression of CBX7 is reduced, which may lead to the lower expression of CBX7 (Lambert et al., 2018). In addition, the regulation of noncoding RNA on gene expression should not be ignored. Studies have shown that long noncoding RNA can regulate gene expression by regulating the binding of transcription factors (Luo et al., 2016; Zhang et al., 2018). Protein ubiquitination and degradation is also an important mechanism used to regulate gene expression (Lan et al., 2016). These are all possible reasons that should be considered and that should be explored further in the future.

We evaluated the expression of members of the CBX family and their associations with the prognosis of patients with LUAD, since molecules with prognostic utility may be more worthy of further study. Because the underlying algorithms used in different datasets may differ slightly, verifying our findings across multiple databases yields more reliable conclusions. We used the UALCAN and KM plotter databases to analyze CBX family members and the prognosis of patients with LUAD, to increase the accuracy of our study. It should also be kept in mind that in survival analysis, numerical variables are generally used, although we should also use both categorical and continuous parameters. Without considering the dual aspects for analysis, the results obtained may be subjected to statistical bias. The two databases we selected applied two different methods for prognostic analysis. Finally, our results showed that CBX3/5/7 levels correlated with prognosis in LUAD patients, albeit in different ways. The expression of CBX3/5 was associated with poor prognosis, while CBX7 showed the opposite trend. What is remarkable about our results is that CBX8 was highly expressed in LUAD, but the higher the expression, the better the prognosis. We speculated that the high expression of CBX8 in lung adenocarcinoma patients may increase the tumors sensitivity to radiotherapy and chemotherapy. Furthermore, compared to normal tissues, the number of lymphocytes infiltrating tumors was increased, a phenomenon which has been confirmed to be associated with increased sensitivity of tumors to immunotherapy and beneficial effects on prognosis of patients (Du et al., 2019:; Tang et al., 2018). Whether there is a similar complex mechanism in the expression of CBX8 and patient prognosis. In addition, the results from the UCLCAN database were not statistically significant (Figure 4P). Nonetheless, to further investigate the exact biological function of CBX8 in larger patient cohorts are needed, which we hope to address in future studies.

Next, we analyzed the genes co-expressed with CBX3/5/7 and performed GO and KEGG Pathway analyses. The results showed that CBX3 was related to the cell cycle and p53 signaling pathways, both of which are closely related to tumorigenesis (Massague, 2004; Sherr and McCormick, 2002). CBX3 has also been associated with the proliferation and migration of LUAD cell lines (Alam et al., 2018). The results showed that CBX5 was associated with the ubiquitin-like protein transferase activity pathway. Ubiquitinated proteins regulate the tumor cell cycle in multiple ways (Nakayama and Nakayama, 2006), and the expression of CBX7 was also associated with the cell cycle in tumors. The frequent reference to the cell cycle is of interest to us. The cell cycle transition is a noteworthy mechanism, because of its direct impact on cell division and survival of tumor cells, which influences the prognosis of patients and is associated with other signaling pathways.

Many recent studies have also reported prognostic signatures constructed by some genes or noncoding RNA in LUAD, glioma, and clear cell renal cell carcinoma (Lin et al., 2020; Xu et al., 2021; Zhang et al., 2020). We also used CBX3/5/7 to construct a prognostic model and confirmed that the signature constructed by these three genes had good prognostic value. The diagnostic value of the model is not satisfactory, and we will continue to optimize our model in the future. The tumor microenvironment is also a hot research topic and is expected to be targeted in new cancer therapies (Wang et al., 2018; Wu and Dai, 2017). Our analysis suggests that CBX3/5/7 may have the ability to modulate immune infiltration. Patients with high expression levels of CBX3/5 had a significantly reduced number of B cells, cytotoxic cells, and CD8^+^ T cells. Conversely, patients with higher levels of CBX7 expression had significantly increased infiltration of all three cell types. For this reason, we performed additional *in vitro* experiments. To date, many studies have reported that several tumor factors influence the secretion of cytokines in tumor cells, which may have an impact on the infiltration of immune cells in the tumor microenvironment. LINC01116 can promote tumor proliferation and neutrophil recruitment through DDX5-mediated regulation of IL-1β in glioma cells; and melanoma-derived factors may alter the maturation and activation of differentiated tissue-resident dendritic cells (Hargadon et al., 2016; Wang et al., 2020). Previous studies determined that IL-6 promotes metastasis of non-small-cell lung cancer via the NF-κB pathway (Liu et al., 2020b). Lung cancer cell lines can secrete cytokines such as IL-6 (see the Supplementary Material), which indicates that lung cancer may regulate the immune microenvironment by secreting cytokines. In addition, other studies have shown that lung cancer cell lines can also secrete IL-8 (Wang et al., 2015b). Recent studies have shown that CBX3 expression is significantly and inversely correlated with to the abundance of tumor-infiltrating lymphocytes, which influences the efficacy of immunotherapy (Lin et al., 2020). These results suggest that CBX molecules may be able to regulate the tumor immune microenvironment, and a potential mechanism involves the regulation of cytokine secretion.

To confirm our findings, we collected primary tissue samples from LUAD patients at our institution. qPCR evaluation of these samples were performed to verify the expression trends of CBX3/5/7. Furthermore, knocking down CBX3/5 expression in A549 cells and overexpressing CBX7 in H1299 cells, we demonstrated that CBX3/5/7 inhibited the proliferation and migration of LUAD cell lines, respectively. CBX3/5 promoted the expression of some cytokines, while CBX7 exerted inhibitory effects. These cytokines have also been shown to play a regulatory role in immune cell recruitment within tumors (Conlon et al., 2019; Ferrari et al., 2019).

Our findings should be considered in the context of the several limitations of the study. We did not study the biological functions of other members of the CBX family, there was no in-depth study of the mechanisms, and no animal experiments were carried out. Our bioinformatics analysis was not sufficiently exhaustive. We did not find any immune-related items among the results of the TOP 10 functional pathways in the enrichment analysis, and thus, we added additional experimental data to investigate immune activity in LUAD. The results obtained by different algorithms also differed, which also prompts the need for additional experimental verification, which will be addressed in future studies investigating pathway enrichment. Further, we did not explore the reasons for the contrasting biological functions among CBX3, CBX5, and CBX7, which will be addressed in future studies.

In summary, we investigated the impact of CBX3/5/7 expression on LUAD including their prognostic value and ability to regulate immune cell infiltration and cell cycle transition. Our results suggest that CBX3/5/7 may be key molecules in LUAD and that these could be used for diagnostic purposes or may provide a rationale to develop new therapies.

## Data Availability

The original contributions presented in the study are included in the article/Supplementary Material, further inquiries can be directed to the corresponding authors.
