# Supplementary figures and images for: Role of the CBX Molecular Family in Lung Adenocarcinoma Tumorigenesis and Immune Infiltration
Source: Front Genet. 2021 Dec 13;12:771062. doi: 10.3389/fgene.2021.771062 (PMC8710700; doi:10.3389/fgene.2021.771062)

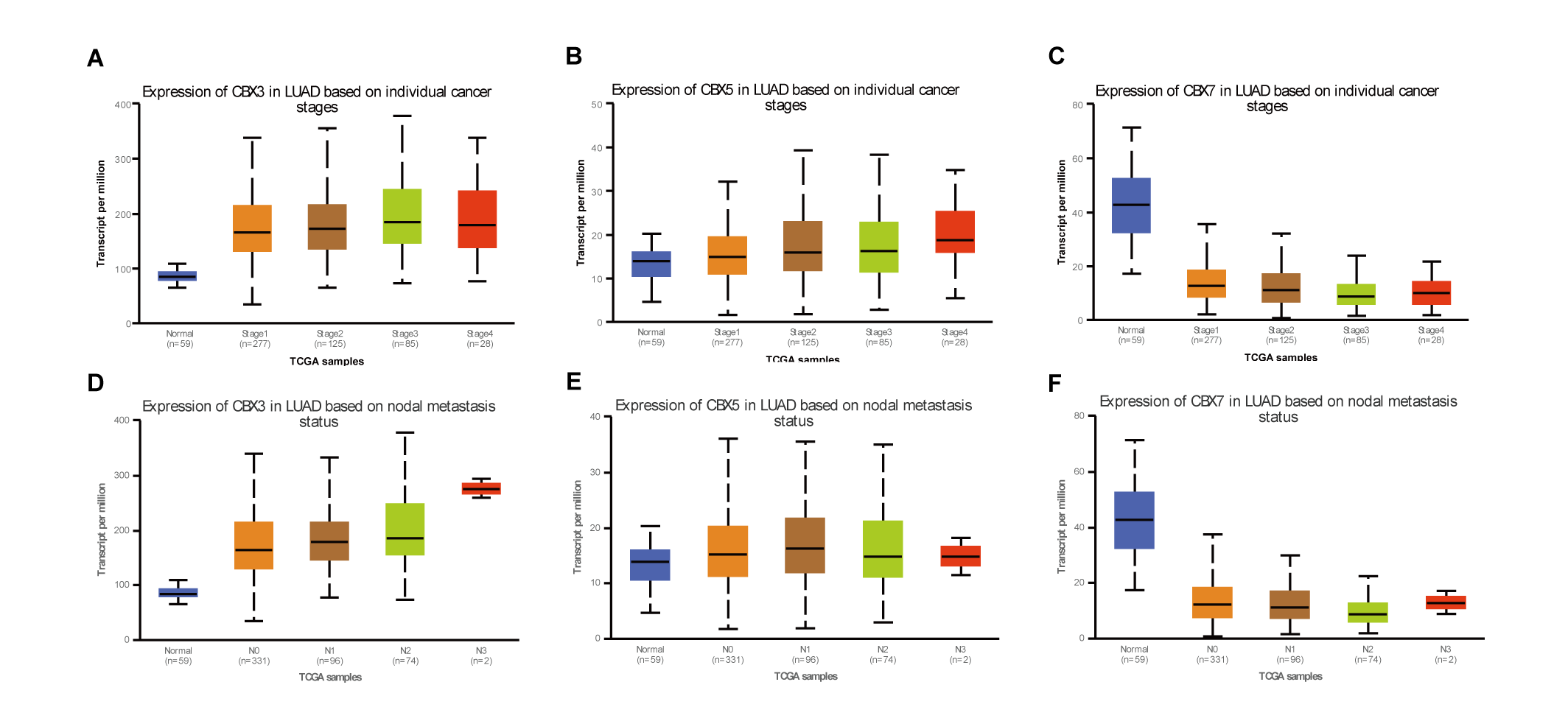

Supplement: Supplementary file 1 [file Image2.TIF]

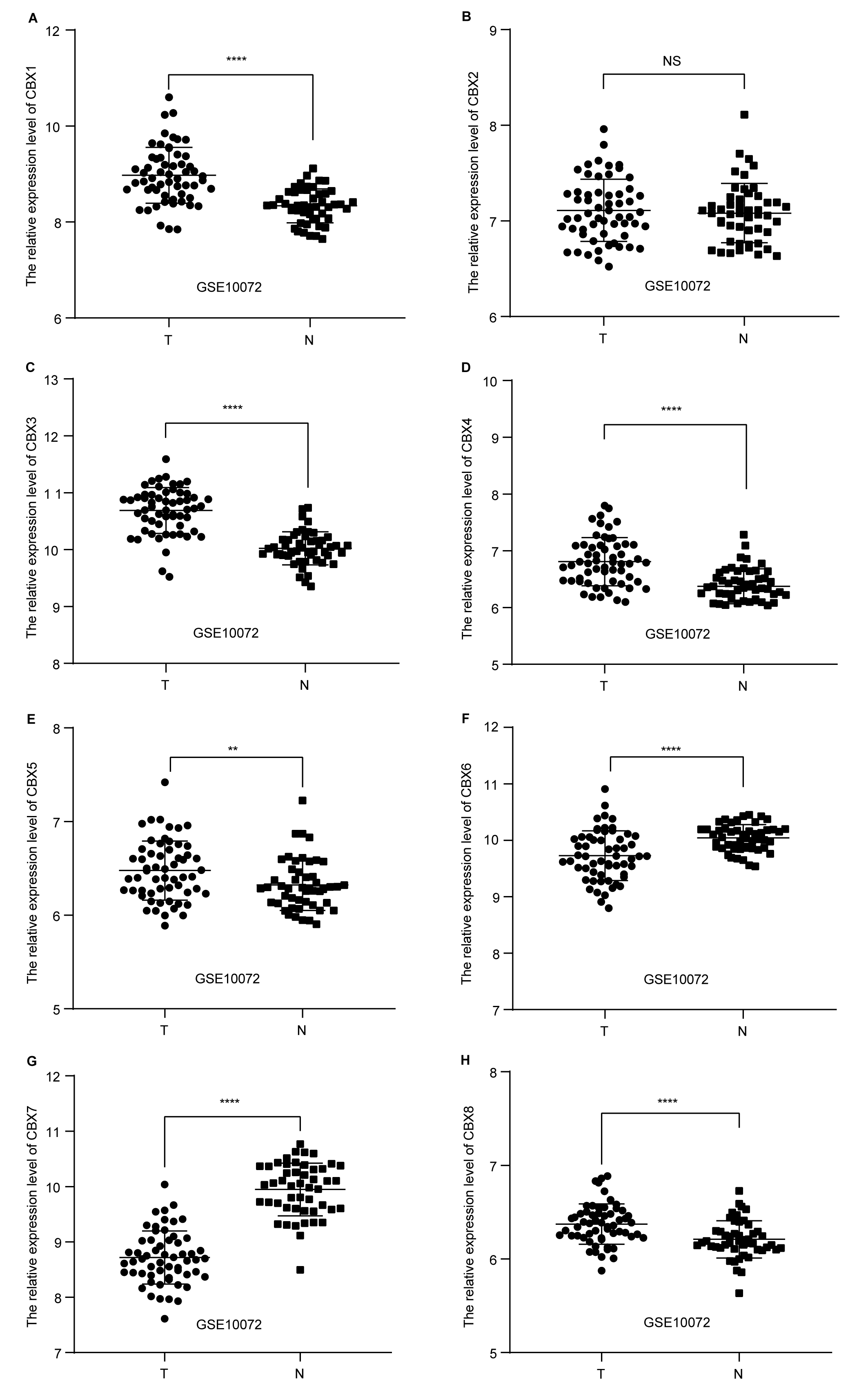

Supplement: Supplementary file 2 [file Image1.TIF]
